# HIV risk among men who have sex with men (MSM) in Nigeria: a potential population for HIV vaccine trial

**DOI:** 10.1186/1742-4690-9-S2-P223

**Published:** 2012-09-13

**Authors:** AO Adeyemi, K Oyediran, KB Issa, A Azeez, A Atobatele, O Fakunle

**Affiliations:** 1 MEASURE, Abuja, Nigeria; 2 Federal Ministry of Health, Abuja, Nigeria; 3 USAID, Abuja, Nigeria; 4 University College Hospital, Abuja, Nigeria

## Background

MSM are at a high risk of HIV. Due to their risk, they are potential population for HIV vaccine trials. Nigeria conducted 2010 Integrated Biological Behavioral Surveillance Survey (IBBSS) among the high risk groups. Unfortunately, MSM had the second highest HIV prevalence after female sex workers, and some with female partners. Thus, HIV prevention among MSM is of great national importance. This study evaluates predictors of their risky behavior which will be useful in enrolling them for future HIV vaccine trials.

## Methods

Secondary analysis of 2010 IBBSS data involving 1545MSM between 18–49years was done. IBSSS involved HIV testing with information collected on sexual/reproductive health indicators. Risky sexual behavior was defined as sex without condom and with more than one male partner. Multivariate logistic regression was used to model predictors of risky sexual behavior.

## Results

Mean age: 25.4±6.0years; HIV prevalence: 17.2%; 12.2% were married to female partners; median anal sex partner was 3. About 31.5% tested and received results for HIV; condom use at last anal sex: 52.1%; sex with girlfriends: 41.9%; 18.3% paid for sex; 12.2% used marijuana; daily alcohol use 26.6% and 33.1% were reached with prevention programs. About 34% MSM engaged in risky sexual behavior. Predictors of risky sexual behavior were use of alcohol OR=2.9 95%CI:1.3-6.1; being away from home for >1month OR=2.5 95%CI:1.3-5.7; anal sex without condom OR=2.9 95%CI:1.6–4.4; sex with ≥3 partners OR=5.6 95%CI:2.1-7.3; while previous STI OR=0.6 95%CI:0.3-0.9; having a female marital partner OR=0.7 95%CI:0.4-0.9; known HIV result prior to survey OR=0.6 95%CI:0.3-0.9 were protective.

## Conclusion

High number of MSM practice risky sexual behavior that is driven by alcohol, multiple male partners and is reduced by previous STI or known HIV result. Lastly, there is a need for innovative and result-oriented behavioral interventions among MSM to reduce their HIV risk.

